# Efficient Certificate-Based Signcryption Secure against Public Key Replacement Attacks and Insider Attacks

**DOI:** 10.1155/2014/295419

**Published:** 2014-05-12

**Authors:** Yang Lu, Jiguo Li

**Affiliations:** College of Computer and Information Engineering, Hohai University, No. 8, Focheng Xi Road, Jiangning District, Nanjing, Jiangsu 211100, China

## Abstract

Signcryption is a useful cryptographic primitive that achieves confidentiality and authentication in an efficient manner. As an extension of signcryption in certificate-based cryptography, certificate-based signcryption preserves the merits of certificate-based cryptography and signcryption simultaneously. In this paper, we present an improved security model of certificate-based signcryption that covers both public key replacement attack and insider security. We show that an existing certificate-based signcryption scheme is insecure in our model. We also propose a new certificate-based signcryption scheme that achieves security against both public key replacement attacks and insider attacks. We prove in the random oracle model that the proposed scheme is chosen-ciphertext secure and existentially unforgeable. Performance analysis shows that the proposed scheme outperforms all the previous certificate-based signcryption schemes in the literature.

## 1. Introduction

Public key cryptography (PKC) is an important technique to realize network and information security. In traditional PKC, a public key infrastructure (PKI) is used to provide an assurance to the users about the relationship between a public key and the holder of the corresponding private key by certificates. However, the need for PKI-supported certificates is considered the main difficulty in the deployment and management of traditional PKC. To simplify the management of the certificates, Shamir [1] introduced the concept of identity-based cryptography (IBC) in which the public key of each user is derived directly from his identity, such as an IP address or an e-mail address, and the corresponding private key is generated by a trusted third party called private key generator (PKG). The main practical benefit of IBC lies in the reduction of need for public key certificates. However, if the KGC becomes dishonest, it can impersonate any user using its knowledge of the user's private key. This is due to the key escrow problem inherent in IBC. In addition, private keys must be sent to the users over secure channels, so private key distribution in IBC becomes a very daunting task.

To fill the gap between traditional PKC and IBC, Al-Riyami and Paterson [2] proposed a new paradigm called certificateless public key cryptography (CL-PKC) in Asiacrypt 2003. CL-PKC eliminates the key escrow problem inherent in IBC. At the same time, it preserves the advantage of IBC which is the absence of certificates and their heavy management overhead. In CL-PKC, a trusted third party called key generating center (KGC) is involved in the process of issuing a partial secret key for each user. The user independently generates its public/private key pair and combines the partial secret key from the KGC with its private key to generate the actual decryption key. By way of contrast to the PKG in IBC, the KGC does not have access to the user's decryption key. Therefore, CL-PKC solves the key escrow problem. However, as partial secret keys must be sent to the users over secure channels, CL-PKC suffers from the distribution problem.

In Eurocrypt 2003, Gentry [3] introduced the notion of certificate-based cryptography (CBC). CBC provides an implicit certification mechanism for a traditional PKI and allows for a periodical update of certificate status. As in traditional PKC, each user in CBC generates his own public/private key pair and requests a certificate from a trusted third party called certifier. The certificate will be pushed only to the owner of the public/private key pair and act as a partial decryption key or a partial signing key. This additional functionality provides an efficient implicit certificate mechanism. For example, in the encryption scenario, a receiver needs both his private key and certificate to decrypt a ciphertext sent to him, while the message sender need not be concerned about the certificate revocation problem. The feature of implicit certification allows us to eliminate third-party queries for the certificate status and simply the public key revocation problem so that CBC does not need infrastructures like CRL and OCSP. Therefore, CBC can be used to construct an efficient PKI requiring fewer infrastructures than the traditional one. Although CBC may be inefficient when a certifier has a large number of users, this problem can be overcome by using* subset covers *[3]. Furthermore, there are no key escrow problem (since the certifier does not know the private keys of users) and key distribution problem (since the certificates need not be kept secret) in CBC.

Table 1 summarizes the comparison of the above cryptosystems.

Since its advent, CBC has attracted great interest in the research community and many schemes have been proposed, including many encryption schemes (e.g., [4–10]) and signature schemes (e.g., [11–16]). As an extension of the signcryption [17] in CBC, Li et al. [18] introduced the concept of certificate-based signcryption (CBSC) that provides the functionalities of encryption and signature simultaneously. As far as we know, there exist three CBSC schemes in the literature so far. In [18], Li et al. proposed the first CBSC scheme based on Chen and Malone-Lee's identity-based signcryption scheme [19]. However, they did not give a formal proof of their security claim. A subsequent paper by Luo et al. [20] proposed the second CBSC scheme alone with a security model of CBSC. Recently, Li et al. [21] proposed a publicly verifiable CBSC scheme that is provably secure in the random oracle model.

*Our Motivations and Contributions.* In this paper, we focus on the construction of a CBSC scheme that resists both the public key replacement attacks and the insider attacks.

Public key replacement attack was first introduced into CL-PKC by Al-Riyami and Paterson [2]. In this attack, an adversary who can replace a user's public key with a value of its choice dupes any other third parties to encrypt messages or verify signatures using a false public key. It seems that this attack does not have effect on CBC since a certifier is employed for issuing a certificate for each user. Unfortunately, some previous research works [13, 16, 22] have demonstrated that it does. In CBC, the certifier does issue the certificates. However, as introduced above, CBC adopts the implicit certificate mechanism so that only the owner of a certificate needs to check the validity of his certificate and others need not be concerned about the status of his certificate. Thus, a malicious user is able to launch the public key replacement attack against an ill-designed certificate-based cryptographic scheme. We observe that Luo et al.'s CBSC scheme [20] is insecure under this attack. The concrete attack can be found in Section 4 of this paper.

Insider security [23] refers to the security against the attacks made by the insider (i.e., the sender or the receiver). It requires that, even if a sender's private key is compromised, an attacker should not be able to designcrypt the message generated by the sender and, even with a receiver's private key, an attacker should not be able to forge a valid signcryption as if generated by the same sender. In contrast to outsider security [23] that refers to the security against the attacks made by the outsider (i.e., any third party except the sender and the receiver), insider security can provide the stronger security for signcryption schemes [24, 25]. Therefore, it has been accepted as a necessary security requirement for a signcryption scheme to achieve. However, none of the previous constructions of CBSC [18, 20, 21] has considered insider security. The previous security models of CBSC [20, 21] only cover the case where the CBSC scheme is attacked by the outsiders. Actually, the public key replacement attack presented in Section 4 also shows that Luo et al.'s CBSC scheme [20] fails in providing insider security.

The main contributions of this paper are as follows.

1. We extend previous works by proposing an improved security model for CBSC that accurately models both the public key replacement attacks and the insider attacks. We show that Luo et al.'s CBSC scheme [20] is insecure in our security model.
2. We develop a new CBSC scheme and formally prove its security in our improved security model. In the random oracle, we prove that the proposed scheme is chosen-ciphertext secure and existentially unforgeable. To the best of our knowledge, it is the first signcryption scheme that achieves security under both the public key replacement attacks and the insider attacks in the certificate-based cryptographic setting. Furthermore, compared with the previous CBSC schemes, our scheme enjoys better performance, especially in the computation efficiency.

*Paper Organization.* The rest of this paper is organized as follows. In the next section, we briefly review some preliminaries required in this paper. In Section 3, we present an improved security model of CBSC. In Section 4, we show that Luo et al.'s CBSC scheme is insecure in our security model. The proposed CBSC scheme is described and analyzed in Section 5. Finally, we draw our conclusions in Section 6.

## 2. Preliminaries

Let *k* be a security parameter and *p* a *k*-bit prime number. Let *G* be an additive cyclic group of prime order *p* and *G*_*T*_ a multiplicative cyclic group of the same order, and let *P* be a generator of *G*. A bilinear pairing is a map *e* : *G* × *G* → *G*_*T*_ satisfying the following properties.

- Bilinearity: for all *P*_1_, *P*_2_ ∈ *G*, and all *a*, *b* ∈ *Z*_p_*, we have *e*(*aP*_1_, *bP*_2_) = *e*(*P*_1_, *P*_2_)^*ab*^.
- Nondegeneracy: *e*(*P*, *P*) ≠ 1.
- Computability: for all *P*_1_, *P*_2_ ∈ *G*, *e*(*P*_1_, *P*_2_) can be efficiently computed.

The security of our CBSC scheme is based on the following hard problems.

**Definition 1**

The computational Diffie-Hellman (CDH) problem in *G* is, given a tuple (*P*, *aP*, *bP*) ∈ *G* ^3^ for unknown *a*, *b* ∈ *Z*_p_*, to compute *a* *bP* ∈ *G*.

**Definition 2 (see [26])**

The bilinear Diffie-Hellman (BDH) problem in (*G*, *G*_*T*_) is, given a tuple (*P*, *aP*, *bP*, *cP*) ∈ *G* ^4^ for unknown *a*, *b*, *c* ∈ *Z*_p_*, to compute *e*(*P*,*P*)^*abc*^ ∈ *G*_*T*_.

**Definition 3 (see [27])**

The collusion attack algorithm with *q*-traitors (*q*-CAA) problem in *G* is given a tuple (*P*, *αP*, (*ω*_1_+*α*)^−1^ *P*,…, (*ω*_*q*_+*α*)^−1^ *P*, *ω*_1_,…, *ω*_*q*_) ∈ *G* ^*q*+2^ × (*Z*_p_*)^*q*^ for unknown *α* ∈ *Z*_p_*, to compute (ω*+*α*)^−1^ *P* for some value ω* ∉ {*ω*_1_,…, *ω*_*q*_}.

**Definition 4 (see [28])**

The modified bilinear Diffie-Hellman inversion for *q*-values (*q*-mBDHI) problem in *G* is given a tuple (*P*, *αP*, (*ω*_1_+*α*)^−1^ *P*,…, (*ω*_*q*_+*α*)^−1^ *P*, *ω*_1_,…, *ω*_*q*_) ∈ *G* ^*q*+2^ × (*Z*_p_*)^*q*^ for unknown *α* ∈ *Z*_p_*, to compute *e*(*P*,*P*)^(ω*+*α*)^−1^^ for some value ω* ∈ *Z*_p_* − {*ω*_1_,…, *ω*_*q*_}.

## 3. Improved Security Model for CBSC Schemes

In this section, we present an improved security model for CBSC that covers both public key replacement attack and insider security. Below, we first briefly review the definition of CBSC.

Formally, a CBSC scheme is specified by the following five algorithms.

- *Setup*(*k*): on input a security parameter *k* ∈ *Z* ^+^, this algorithm generates a master key *msk* and a list of public parameters *params*. This algorithm is performed by a certifier. After the algorithm is performed, the certifier publishes *params* and keeps *msk* secret.
- *UserKeyGen*(*params*): on input the public parameters *params*, this algorithm generates a private key and public key pair (*SK*_*U*_, *PK*_*U*_) for a user with identity *id*_*U*_.
- *CertGen*(*params*, *msk*, *id*_*U*_, *PK*_*U*_): on input the public parameters *params*, the master key *msk*, a user's identity *id*_*U*_, and public key *PK*_*U*_, this algorithm generates a certificate *Cert*_*U*_. This algorithm is performed by a certifier. After this algorithm is performed, the certifier sends the certificate *Cert*_*U*_ to the user *id*_*U*_ via an open channel.
- *Signcrypt*(*params*, *M*, *id*_*S*_, *PK*_*S*_, *SK*_*S*_, *Cert*_*S*_, *id*_*R*_, *PK*_*R*_): on input the public parameters *params*, a sender's identity *id*_*S*_, public key *PK*_*S*_, private key *SK*_*S*_ and certificate *Cert*_*S*_, a receiver's identity *id*_*R*_, and public key *PK*_*R*_, this algorithm generates a ciphertext *σ*.
- *Designcrypt*(*params*, *σ*, *id*_*R*_, *PK*_*R*_, *SK*_*R*_, *Cert*_*R*_, *id*_*S*_, *PK*_*S*_): on input the public parameters *params*, a ciphertext *σ*, the receiver's identity *id*_*R*_, public key *PK*_*R*_, private key *SK*_*R*_ and certificate *Cert*_*R*_, the sender's identity *id*_*S*_, and public key *PK*_*S*_, this algorithm outputs either a plaintext *M* or a special symbol ⊥ indicating a designcryption failure.

As introduced in [3], the adversaries against a certificate-based cryptographic scheme should be divided into two types: Type I and Type II. Type I adversary (denoted by *A*_*I*_) models an uncertified user while Type II adversary (denoted by *A*_*II*_) models an honest-but-curious certifier who is equipped with the master key. In order to capture public key replacement attack, the Type I adversary *A*_*I*_ in our CBSC security model is allowed to replace any user's public key. Note that the Type II adversary *A*_*II*_ should not be allowed to make public key replacement attacks; otherwise, it may trivially break the security of a CBSC scheme using a man-in-the-middle attack.

A CBSC scheme should satisfy both confidentiality (i.e., indistinguishability against adaptive chosen-ciphertext attacks (IND-CBSC-CCA2)) and unforgeability (i.e., existential unforgeability against adaptive chosen-messages attacks (EUF-CBSC-CMA)).

The confidentiality security of a CBSC scheme is defined via the following two games: “*IND-CBSC-CCA2 Game-I*” and “*IND-CBSC-CCA2 Game-II,*” in which a Type I adversary *A*_*I*_ and a Type II adversary *A*_*II*_ interact with a challenger, respectively.

*IND-CBSC-CCA2 Game-I. *This game is played between *A*_*I*_ and a challenger. 

*Setup.* The challenger runs the algorithm* Setup*(*k*) to generate *msk* and *params*. It then returns *params* to *A*_*I*_ and keeps *msk* to itself. 

*Phase 1.* In this phase, *A*_*I*_ makes requests to the following oracles adaptively.

- *O* ^*CreateUser*^: on input an identity *id*_*U*_, if *id*_*U*_ has already been created; the challenger outputs the current public key *PK*_*U*_ associated with *id*_*U*_. Otherwise, it performs the algorithm* UserKeyGen*(*params*) to generate a private/public key pair (*SK*_*U*_, *PK*_*U*_), inserts (*id*_*U*_, *PK*_*U*_, *SK*_*U*_) into a list, and outputs *PK*_*U*_. In this case, *id*_*U*_ is said to be created. We assume that other oracles defined below only respond to an identity which has been created.
- *O* ^*ReplacePublicKey*^: on input an identity *id*_*U*_ and a value *PK*_*U*_′, the challenger replaces the current public key of the identity *id*_*U*_ with *PK*_*U*_′. Note that the current value of a user's public key is used by the challenger in any computations or responses to *A*_*I*_'s requests. This oracle models the ability of a Type I adversary to convince a legitimate user to use a false public key and thus enables our security model to capture the public key replacement attacks attempted by the Type I adversary *A*_*I*_.
- *O* ^*GenerateCertificate*^: on input an identity *id*_*U*_, the challenger responds with a certificate* Cert*_*U*_ by running the algorithm* CertGen*(*params*, *msk*, *id*_*U*_, *PK*_*U*_).
- *O* ^*ExtractPrivateKey*^: on input an identity *id*_*U*_, the challenger responds with a private key *SK*_*U*_. Here, *A*_*I*_ is disallowed to query this oracle on any identity for which the public key has been replaced. This restriction is imposed due to the fact that it is unreasonable to expect the challenger to be able to provide a private key of a user for which it does not know the private key.
- *O* ^*Signcryption*^: on input a message *M*, a sender's identity *id*_*S*_, and a receiver's identity *id*_*R*_, the challenger responds with *σ* = *Signcrypt*(*params*, *M*, *id*_*S*_, *PK*_*S*_, *SK*_*S*_, *Cert*_*S*_, *id*_*R*_, *PK*_*R*_). Note that it is possible that the challenger is not aware of the sender's private key if the associated public key has been replaced. In this case, we require *A*_*I*_ to provide it. In addition, we do not consider attacks targeting ciphertexts where the identities of the sender and receiver are the same. So, we disallow queries where *id*_*S*_ = *id*_*R*_.
- *O* ^*Designcryption*^: on input a ciphertext *σ*, a sender's identity *id*_*S*_, and a receiver's identity *id*_*R*_, the challenger responds with the result of* Designcrypt*(*params*, *σ*, *id*_*R*_, *PK*_*R*_, *SK*_*R*_, *Cert*_*R*_, *id*_*S*_, *PK*_*S*_). Note that it is possible that the challenger is not aware of the receiver's private key if the associated public key has been replaced. In this case, we require *A*_*I*_ to provide it. Again, we disallow queries where *id*_*S*_ = *id*_*R*_.

*Challenge.* Once *A*_*I*_ decides that Phase 1 is over, it outputs two equal-length messages (*M*_0_, *M*_1_) and two distinct identities (*id*_S_*, *id*_R_*). The challenger picks a random bit *b*, computes σ* = *Signcrypt*(*params*, *M*_*b*_, *id*_S_*, *PK*_S_*, *SK*_S_*, *Cert*_S_*, *id*_R_*, *PK*_R_*), and returns σ* as the challenge ciphertext to *A*_*I*_. 

*Phase 2.* In this phase, *A*_*I*_ continues to issues queries as in Phase 1. 

*Guess.* Finally, *A*_*I*_ outputs a guess *b*′ ∈ {0,1}. We say that *A*_*I*_ wins the game if *b* = *b*′ and the following conditions are simultaneously satisfied: (1)  *A*_*I*_ cannot query *O* ^*GenerateCertificate*^ on the identity *id*_R_* at any point; (2)  *A*_*I*_ cannot query *O* ^*ExtractPrivateKey*^ on an identity if the corresponding public key has been replaced; (3) in Phase 2, *A*_*I*_ cannot query *O* ^*Designcryption*^ on (σ*, *id*_S_*, *id*_R_*) unless the public key of the sender *id*_S_* or that of the receiver *id*_R_* has been replaced after the challenge was issued. We define *A*_*I*_'s advantage in this game to be 2|Pr{*b* = *b*′} − 1/2|. 

*IND-CBSC-CCA2 Game-II. *This game is played between *A*_*II*_ and a challenger. 

*Setup.* The challenger runs the algorithm* Setup*(*k*) to generate *msk* and *params*. It then returns *params* and *msk* to *A*_*II*_. 

*Phase 1.* In this phase, *A*_*II*_ adaptively asks a polynomial bounded number of queries as in* IND-CBSC-CCA2 Game-I*. The only restriction is that *A*_*II*_ cannot replace public keys of any users. In addition, *A*_*II*_ need not make any queries to *O* ^*GenerateCertificate*^ since it can compute the certificates for any identities by itself with the master key *msk*. 

*Challenge*. Once *A*_*II*_ decides that Phase 1 is over, it outputs two equal-length messages (*M*_0_, *M*_1_) and two distinct identities (*id*_S_*, *id*_R_*). The challenger picks a random bit *b*, computes σ* = *Signcrypt*(*params*, *M*_*b*_, *id*_S_*, *PK*_S_*, *SK*_S_*, *Cert*_S_*, *id*_R_*, *PK*_R_*), and returns σ* as the challenge ciphertext to *A*_*II*_. 

*Phase 2.* In this phase, *A*_*II*_ continues to issue queries as in Phase 1. 

*Guess.* Finally, *A*_*II*_ outputs a guess *b*′ ∈ {0,1}. We say that *A*_*II*_ wins the game if *b* = *b*′ and the following two conditions are both satisfied: (1)  *A*_*II*_ cannot query *O* ^*ExtractPrivateKey*^ on the identity *id*_R_* at any point; (2)  *A*_*II*_ cannot query *O* ^*Designcryption*^ on (σ*, *id*_S_*, *id*_R_*) in Phase 2. We define *A*_*II*_'s advantage in this game to be 2|Pr{*b* = *b*′} − 1/2|.

**Definition 5**

A CBSC scheme is said to be IND-CBSC-CCA2 secure if no probabilistic polynomial time (PPT) adversary has nonnegligible advantage in the above two games.

**Remark 6**

The oracle *O* ^*ReplacePublicKey*^ defined in the game* IND-CBSC-CCA2 Game-I* models the ability of a Type I adversary to convince a legitimate user to use a false public key. It enables our security model to capture the public key replacement attacks attempted by the Type I adversary *A*_*I*_.

**Remark 7**

The adversary in the above definition of message confidentiality is allowed to be challenged on a ciphertext generated using a corrupted sender's private key and certificate. This condition corresponds to the stringent requirement of insider security for confidentiality of signcryption [23]. This means that our security model ensures that the confidentiality of signcryption is preserved even if a sender's private key is corrupted.

The unforgeability security of a CBSC scheme is defined via the following two games: “*EUF-CBSC-CMA Game-I*” and “*EUF-CBSC-CMA Game-II,*” in which a Type I adversary *A*_*I*_ and a Type II adversary *A*_*II*_ interact with a challenger, respectively.

*EUF-CBSC-CMA Game-I. *This game is played between *A*_*I*_ and a challenger. 

*Setup.* The challenger runs the algorithm* Setup*(*k*) to generate *msk* and *params*. It then returns *params* to *A*_*I*_ and keeps *msk* to itself. 

*Query.* In this phase, *A*_*I*_ can adaptively ask a polynomial bounded number of queries as in the game* IND-CBSC-CCA2 Game-I*. 

*Forge.* Finally, *A*_*I*_ outputs a forgery (σ*, *id*_S_*, *id*_R_*). We say that *A*_*I*_ wins the game if the result of* Designcrypt*(*params*, σ*, *id*_R_*, *PK*_R_*, *SK*_R_*, *Cert*_R_*, *id*_S_*, *PK*_S_*) is not the ⊥ symbol and the following conditions are simultaneously satisfied: (1)  *A*_*I*_ cannot query *O* ^*GenerateCertificate*^ on the identity *id*_S_* at any point; (2)  *A*_*I*_ cannot query *O* ^*ExtractPrivateKey*^ on an identity if the corresponding public key has been replaced; (3)  σ* is not the output of any *O* ^*Signcryption*^ query on (M*, *id*_S_*, *id*_R_*), where M* is a message. We define *A*_*I*_'s advantage in this game to be the probability that it wins the game. 

*EUF-CBSC-CMA Game-II. *This game is played between *A*_*II*_ and a challenger. 

*Setup.* The challenger runs the algorithm* Setup*(*k*) to generate *msk* and *params*. It then returns *params* and *msk* to *A*_*II*_. 

*Query.* In this phase, *A*_*II*_ can adaptively ask a polynomial bounded number of queries as in the game* IND-CBSC-CCA2 Game-II*. 

*Forge.* Finally, *A*_*II*_ outputs a forgery (σ*, *id*_S_*, *id*_R_*). We say that *A*_*II*_ wins the game if the result of* Designcrypt*(*params*, σ*, *id*_R_*, *PK*_R_*, *SK*_R_*, *Cert*_R_*, *id*_S_*, *PK*_S_*) is not the ⊥ symbol and the following conditions are simultaneously satisfied: (1)  *A*_*II*_ cannot query *O* ^*ExtractPrivateKey*^ on the identity *id*_S_*; (2)  σ* is not the output of any *O* ^*Signcryption*^ query on (M*, *id*_S_*, *id*_R_*), where M* is a message. We define *A*_*II*_'s advantage in this game to be the probability that it wins the game.

**Definition 8**

A CBSC scheme is said to be EUF-CBSC-CMA secure if no PPT adversary has nonnegligible advantage in the above two games.

**Remark 9**

The adversary in the above definition of signature unforgeability may output a ciphertext generated using a corrupted receiver's private key and certificate. Again, this condition corresponds to the stringent requirement of insider security for unforgeability of signcryption [23]. Hence, our security model also ensures that the unforgeability of signcryption is preserved even if a receiver's private key is corrupted.

## 4. Cryptanalysis of Luo et al.'s CBSC Scheme

In this section, we give the review and attack of Luo et al.'s CBSC scheme [20].

### 4.1. Review of Luo et al.'s CBSC Scheme

Luo et al.'s CBSC scheme consists of the following six algorithms.

- *Setup:* given a security parameter *k*, the certifier performs as follows: generate two cyclic groups *G* and *G*_*T*_ of prime order *p* such that there exists a bilinear pairing map *e* : *G* × *G* → *G*_*T*_; select a random element *s* ∈ *Z*_p_* and a random generator *P* ∈ *G*, and compute *P*_pub_ = *sP*; select four hash functions *H*_1_ : {0,1}^*n*^ × *G* → *G*, *H*_2_ : {0,1}^*n*^ × *G* × *G* → *G*, *H*_3_ : *G* × *G* × {0,1}^*n*^ → *Z*_p_*, and *H*_4_ : *G*_*T*_ → {0,1}^*n*^; set the public parameters *params* = {*p*, *G*, *G*_*T*_, *e*, *n*, *P*, *P*_pub_, *H*_1_, *H*_2_, *H*_3_, *H*_4_} and the master key *msk* = *s*.
- *UserKeyGen:* given *params*, a user with identity *id*_*U*_ ∈ {0, 1}^*n*^ chooses a random *x*_*U*_ ∈ *Z*_p_* as his private key *SK*_*U*_ and then computes his public key *PK*_*U*_ = *x*_*U*_ *P*.
- *CertGen:* to generate a certificate for the user with identity *id*_*U*_ and public key *PK*_*U*_, the certifier computes *Q*_*U*_ = *H*_1_(*id*_*U*_, *PK*_*U*_) and outputs the certificate *Cert*_*U*_ = *sQ*_*U*_.
- *Sender Signcrypt:* to send a message *M* ∈ {0, 1}^*n*^ to the receiver *id*_*R*_, the sender *id*_*S*_ does the following: randomly choose *r* ∈ *Z*_p_* and compute *R* = *rP* and *T* = *H*_2_(*id*_*S*_, *PK*_*S*_, *R*); compute *h* = *H*_3_(*R*, *S*, *M*) and *V* = *r* ^−1^(*Cert*_*S*_ + *SK*_*S*_ · *T* + *h* · *P*_pub_); compute *W* = *e*(*PK*_*S*_, *PK*_*R*_)^*r*^ and then *C* = *M* ⊕ *H*_4_(*W*); set the ciphertext *σ* = (*C*, *R*, *V*).
- *Receiver Decrypt:* when receiving a ciphertext *σ* = (*C*, *R*, *V*) from the sender *id*_*S*_, the receiver *id*_*R*_ does the following: compute *M* = *C* ⊕ *H*_4_(*W*) where *W* = *e*(*R*, *SK*_*R*_ · *PK*_*S*_); forward the message *M* and signature (*R*, *V*) to the algorithm* Receiver Verify*.
- *Receiver Verify:* to verify the sender *id*_*S*_'s signature (*R*, *V*) on the message *M*, the receiver *id*_*R*_ does the following: compute *S* = *H*_2_(*id*_*S*_, *PK*_*S*_, *R*) and *h* = *H*_3_(*R*, *S*, *M*); check whether *e*(*R*, *V*) = *e*(*P*_pub_, *Q*_*S*_)*e*(*PK*_*S*_, *S*)*e*(*P*, *P*_pub_)^*h*^. If the check holds, output *M*; otherwise, output ⊥.

### 4.2. Attack on Luo et al.'s CBSC Scheme

A Type I adversary who is capable of replacing any user's public key can forge a valid signcryption on any message *M* from *id*_*S*_ to *id*_*R*_ by performing the following steps.

1. Replace the sender *id*_*S*_'s public key with *PK*_*S*_′ = *x*_*S*_′*P*_pub_, where *x*_*S*_′ is a random value chosen from *Z*_p_*.
2. Choose a random value *r*′ ∈ *Z*_p_* and compute *R*′ = *r*′*P*_pub_ and *T*′ = *H*_2_(*id*_*S*_, *PK*_*S*_′, *R*′).
3. Choose a random message *M* ∈ {0,1}^*n*^ and compute *V*′ = *r* ^′−1^(*Q*_*S*_ + *x*_*S*_′*T*′ + *h*′*P*), where *Q*_*S*_ = *H*_1_(*id*_*S*_, *PK*_*S*_′) and *h*′ = *H*_3_(*R*′, *T*′, *M*).
4. Randomly choose *C*′ ∈ {0,1}^*n*^ and set *σ*′ = (*C*′, *R*′, *V*′) as the signcryption of the message *M*. Note that if the adversary has corrupted the receiver *id*_*R*_'s private key *SK*_*R*_, it can compute *C*′ = *M* ⊕ *H*_4_(*W*′), where *W*′ = *e*(*R*′, *SK*_*R*_, *PK*_*S*_′).

The ciphertext *σ*′ = (*C*′, *R*′, *V*′) passes the verification test as shown below:

(1) e(Ppub,QS)e(PKS′,T′)e(P,Ppub)h′ =e(Ppub,QS+xS′T′+h′P) =e(r′Ppub,r′−1(QS+xS′T′+h′P)) =e(R′,V′).

This proves that the forged signcryption is valid.

Note that Luo et al.'s scheme also doses not resist insider attacks since the adversary can forge a valid signcryption using the corrupted receiver *id*_*R*_'s private key in the step (4).

## 5. Our Proposed CBSC Scheme

### 5.1. Description of the Scheme

Our CBSC scheme is constructed from the certificate-based encryption scheme proposed by Lu et al. [8]. It consists of the following five algorithms.

- *Setup*(*k*): given a security parameter *k*, the certifier performs the following: generate two cyclic groups *G* and *G*_*T*_ of a *k*-bit prime order *p* such that there exists a bilinear pairing map *e* : *G* × *G* → *G*_*T*_; choose two random generators *P*, *Q* ∈ *G* and compute *g* = *e*(*P*, *Q*); choose a random element *α* ∈ *Z*_p_* and set *P*_pub  _ = *αP*; select three hash functions *H*_1_ : {0,1}* × *G*_*T*_ → *Z*_p_*, *H*_2_ : *G*_*T*_ × *G*_*T*_ → {0,1}^*n*^ and *H*_3_ : {0,1}* → *Z*_p_*, where *n* is the bit-length of the message to be signcrypted; set the public parameters *params* = {*p*, *G*, *G*_*T*_, *e*, *n*, *P*, *Q*, *P*_pub_, *g*, *H*_1_, *H*_2_, *H*_3_} and the master key *msk* = *α*.
- *UserKeyGen*(*params*): given *params*, a user with identity *id*_*U*_ ∈ {0,1}* chooses a random *x*_*U*_ ∈ *Z*_p_* as his private key *SK*_*U*_ and then computes his public key *PK*_*U*_ = *g* ^*x*_*U*_^.
- *CertGen*(*params*, *msk*, *id*_*U*_, *PK*_*U*_): to generate a certificate for a user with identity *id*_*U*_ and public key *PK*_*U*_, the certifier computes *Cert*_*U*_ = (*H*_1_(*id*_*U*_,*PK*_*U*_)+*α*)^−1^ *Q*. The user *id*_*U*_ can check the validness of the certificate *Cert*_*U*_ by verifying whether *e*(*H*_1_(*id*_*U*_, *PK*_*U*_)*P* + *P*_pub_, *Cert*_*U*_) = *g*.
- *Signcrypt*(*params*, *M*, *id*_*S*_, *PK*_*S*_, *SK*_*S*_, *Cert*_*S*_, *id*_*R*_, *PK*_*R*_): to send a message *M* ∈ {0,1}^*n*^ to the receiver *id*_*R*_, the sender *id*_*S*_ does the following: randomly choose *r* ∈ *Z*_p_* and compute *R*_1_ = *g* ^*r*^ and *R*_2_ = (*PK*_*R*_)^*r*^; compute *U* = *r*(*H*_1_(*id*_*R*_, *PK*_*R*_)*P* + *P*_pub_) and *C* = *M* ⊕ *H*_2_(*R*_1_, *R*_2_); compute *V* = (*h* · *SK*_*S*_ + *r*) · *Cert*_*S*_, where *h* = *H*_3_(*M*, *U*, *R*_1_, *R*_2_, *id*_*S*_, *PK*_*S*_, *id*_*R*_, *PK*_*R*_); set the ciphertext *σ* = (*C*, *U*, *V*).
- *Designcrypt*(*params*, *σ*, *id*_*R*_, *PK*_*R*_, *SK*_*R*_, *Cert*_*R*_, *id*_*S*_, *PK*_*S*_): to designcrypt a ciphertext *σ* = (*C*, *U*, *V*) from the sender *id*_*S*_, the receiver *id*_*R*_ does the following: compute *R*_1_ = *e*(*U*, *Cert*_*R*_) and *R*_2_ = *e*(*U*, *Cert*_*R*_)^*SK*_*R*_^; compute *M* = *C* ⊕ *H*_2_(*R*_1_, *R*_2_) and then check whether *e*(*H*_1_(*id*_*S*_, *PK*_*S*_)*P* + *P*_pub_, *V*)(*PK*_*S*_)^−*h*^ = *R*_1_, where *h* = *H*_3_(*M*, *U*, *R*_1_, *R*_2_, *id*_*S*_, *PK*_*S*_, *id*_*R*_, *PK*_*R*_). If the check holds, output *M*; otherwise, output ⊥.

The consistency of our scheme can be easily verified by the following equalities:

(1) *e*(*U*, *Cert*_*R*_) = *e*(*r*(*H*_1_(*id*_*R*_, *PK*_*R*_)*P* + *P*_pub_), (*H*_1_(*id*_*R*_, *PK*_*R*_) + *α*)^−1^ *Q*) = *e*(*P*, *Q*)^*r*^ = *g* ^*r*^;
(2) *e*(*U*,*Cert*_*R*_)^*SK*_*R*_^ = (*g* ^*r*^)^*SK*_*R*_^ = (*PK*_*R*_)^*r*^;
(3) (2) e(H1(idS,PKS)P+Ppub,V)(PKS)−h =e((H1(idS,PKS)+α)−1P,(h·SKS+r)·CertS)  ·(PKS)−h =e(P,(h·SKS+  r)Q)·(PKS)−h =e(P,Q)r=R1.

### 5.2. Security Proof

**Theorem 10**

The CBSC scheme above is IND-CBSC-CCA2 secure under the hardness of the q-mBDHI and BDH problems in the random oracle model.

This theorem can be proved by combining the following two lemmas.

**Lemma 11**

If a Type I adversary *A*_*I*_ has advantage *ε* against our CBSC scheme when asking at most *q*_*cu*_ to *O* ^CreateUser^ queries, *q*_*sc*_ queries to *O* ^*Signcryption*^, *q*_*dsc*_ queries to *O* ^*Designcryption*^, and *q*_*i*_ queries to random oracles *H*_1_ ~ *H*_3_, then there exists an algorithm *B* to solve the (*q*_1_ − 1)-mBDHI problem with advantage

(3) ε′≥εq1(q2+2q3+2qsc)(1−qscq2+2q3+2qsc2k) ×(1−qdsc2k).

**Proof**

Assume that B is given a random *q*-mBDHI instance (*P*, *αP*, (*ω*_1_ + *α*)^−1^ *P*,…, (*ω*_*q*_ + *α*)^−1^ *P*, *ω*_1_,…, *ω*_*q*_), where *q* = *q*_1_ − 1. B interacts with *A*_*I*_ as follows.

In the setup phase, B randomly chooses *t* ∈ *Z*_p_* and sets *P*_pub_ = *αP*, *Q* = *tP*, and *g* = *e*(*P*, *Q*). Furthermore, it randomly chooses a value ω* ∈ *Z*_p_* such that ω* ∉ {*ω*_1_,…, *ω*_*q*_} and an index *θ* ∈ [1, *q*_1_]. Then, B starts* IND-CBSC-CCA2 Game-I* by supplying *A*_*I*_ with *params* = {*p*, *G*, *G*_*T*_, *e*, *n*, *P*, *Q*, *P*_pub_, *g*, *H*_1_, *H*_2_, *H*_3_}, where *H*_1_ ~ *H*_3_ are random oracles controlled by B.  *A*_*I*_ can make queries on these random oracles at any time during the game. Note that the corresponding master key is *msk* = *α* which is unknown to B.

Now, B starts to respond to various queries as follows:

*H*_1_ *Queries.* We assume that *q*_1_ queries to *H*_1_ are distinct. B maintains a list H_1_List of tuples 〈*id*_*i*_, *PK*_*i*_, *h*_1,*i*_, *Cert*_*i*_〉. On input (*id*_*i*_, *PK*_*i*_), B does the following.

1. If (*id*_*i*_, *PK*_*i*_) already appears on H_1_List in a tuple 〈*id*_*i*_, *PK*_*i*_, *h*_1,*i*_, *Cert*_*i*_〉, then B returns *h*_1,*i*_ to *A*_*I*_.
2. Else if the query is on the *θ*th distinct (*id*_*θ*_, *PK*_*θ*_), then B inserts 〈*id*_*θ*_, *PK*_*θ*_, ω*, ⊥〉 into H_1_List and returns *h*_1,*θ*_ = ω* to *A*_*I*_. Note that the certificate for the identity *id*_*θ*_ is *Cert*_*θ*_ = *t*(ω* + *α*)^−1^ *P* which is unknown to B.
3. Else B sets *h*_1,*i*_ to be *ω*_*j*_  (*j* ∈ [1, *q*]) which has not been used and computes *Cert*_*i*_ = *t*(*ω*_*j*_ + *α*)^−1^ *P*. It then inserts 〈*id*_*i*_, *PK*_*i*_, *h*_1,*i*_, *Cert*_*i*_〉 into H_1_List and returns *h*_1,*i*_.

*H*_2_ *Queries. *B maintains a list H_2_List of tuples 〈*R*_1_, *R*_2_, *h*_2_〉. On input (*R*_1_, *R*_2_), B does the following.

1. If (*R*_1_, *R*_2_) already appears on H_2_List in a tuple 〈*R*_1_, *R*_2_, *h*_2_〉, B returns *h*_2_ to *A*_*I*_.
2. Otherwise, it returns a random *h*_2_ ∈ {0, 1}^*n*^ and inserts 〈*R*_1_, *R*_2_, *h*_2_〉 into H_2_List.

*H*_3_ *Queries*. B maintains a list H_3_List of tuples 〈*M*, *U*, *R*_1_, *R*_2_, *id*_*S*_, *PK*_*S*_, *id*_*R*_, *PK*_*R*_, *h*_3_, *C*〉. On input (*M*, *U*, *R*_1_, *R*_2_, *id*_*S*_, *PK*_*S*_, *id*_*R*_, *PK*_*R*_), B does the following.

1. If (*M*, *U*, *R*_1_, *R*_2_, *id*_*S*_, *PK*_*S*_, *id*_*R*_, *PK*_*R*_) already appears on H_3_List in a tuple 〈*M*, *U*, *R*_1_, *R*_2_, *id*_*S*_, *PK*_*S*_, *id*_*R*_, *PK*_*R*_, *h*_3_, *C*〉, B returns *h*_3_ to *A*_*I*_.
2. Otherwise, it returns a random *h*_3_ ∈ *Z*_p_* to *A*_*I*_. To anticipate possible subsequent queries to *O* ^Designcryption^, it additionally simulates the random oracle *H*_2_ on its own to obtain *h*_2_ = *H*_2_(*R*_1_, *R*_2_) and then inserts 〈*M*, *U*, *R*_1_, *R*_2_, *id*_*S*_, *PK*_*S*_, *id*_*R*_, *PK*_*R*_, *h*_3_, *C* = *M* ⊕ *h*_2_〉 into H_3_List.

*O* ^*CreateUser*^ *Queries*. B maintains a list KeyList of tuples 〈*id*_*i*_, *PK*_*i*_, *SK*_*i*_, flag_*i*_〉 which is initially empty. On input (*id*_*i*_), B does the following.

1. If *id*_*i*_ already appears on KeyList in a tuple 〈*id*_*i*_, *PK*_*i*_, *SK*_*i*_, flag_*i*_〉, B returns *PK*_*i*_ to *A*_*I*_ directly.
2. Otherwise, B randomly chooses *x*_*i*_ ∈ *Z*_p_* as the private key *SK*_*i*_ for the identity *id*_*i*_ and computes the corresponding public key as *PK*_*i*_ = *g* ^*x*_*i*_^. It then inserts 〈*id*_*i*_, *PK*_*i*_, *SK*_*i*_, 0〉 into KeyList and returns *PK*_*i*_ to *A*_*I*_.

*O* ^*ReplacePublicKeyr*^ *Queries*. On input (*id*_*i*_, *PK*_*i*_′), B searches *id*_*i*_ in KeyList to find a tuple 〈*id*_*i*_, *PK*_*i*_, *SK*_*i*_, flag_*i*_〉 and updates the tuple with 〈*id*_*i*_, *PK*_*i*_′, *SK*_*i*_, 1〉.

*O* ^*ExtractPrivateKey*^ *Queries*. On input (*id*_*i*_), B searches *id*_*i*_ in KeyList to find a tuple 〈*id*_*i*_, *PK*_*i*_, *SK*_*i*_, flag_*i*_〉. If flag_*i*_ = 0, it returns *SK*_*i*_ to *A*_*I*_; otherwise, it rejects this query.

*O* ^*GenerateCertificate*^ *Queries*. On input (*id*_*i*_), B does the following.

1. If (*id*_*i*_, *PK*_*i*_) = (*id*_*θ*_, *PK*_*θ*_), then B aborts.
2. Otherwise, B searches *id*_*i*_ in H_1_List to find a tuple 〈*id*_*i*_, *PK*_*i*_, *h*_1,*i*_, *Cert*_*i*_〉 and then returns *Cert*_*i*_ to *A*_*I*_. If H_1_List does not contain such a tuple, B queries *H*_1_ on (*id*_*i*_, *PK*_*i*_) first.

*O* ^*Signcryption*^ *Queries*. On input (*M*, *id*_*S*_, *id*_*R*_), B performs as follows.

1. If (*id*_*S*_, *PK*_*S*_)≠(*id*_*θ*_, *PK*_*θ*_), B can answer the query according to the specification of the algorithm* Signcrypt* since it knows the sender *id*_*S*_'s private key and certificate.
2. Otherwise, B randomly chooses *r*, *h*_3_ ∈ *Z*_p_*, *h*_2_ ∈ {0, 1}^*n*^ and sets *U* = *r*(*H*_1_(*id*_*θ*_, *PK*_*θ*_)*P* + *P*_pub_) − *h*_3_ *SK*_*θ*_(*H*_1_(*id*_*R*_, *PK*_*R*_)*P* + *P*_pub_), *V* = *r* *Cert*_*R*_, *C* = *M* ⊕ *h*_2_, *R*_1_ = *e*(*U*, *Cert*_*R*_), and *R*_2_ = *e*(*U*,*Cert*_*R*_)^*SK*_*R*_^, respectively. It is easy to verify that *e*(*H*_1_(*id*_*θ*_, *PK*_*θ*_)*P* + *P*_pub_, *V*)·(*PK*_*θ*_)^−*h*_3_^ = *e*(*U*, *Cert*_*R*_). Then, B inserts 〈*R*_1_, *R*_2_, *h*_2_〉 and 〈*M*, *U*, *R*_1_, *R*_2_, *id*_*θ*_, *PK*_*θ*_, *id*_*R*_, *PK*_*R*_, *h*_3_, *C*〉 into H_2_List and H_3_List, respectively, and returns the ciphertext *σ* = (*C*, *U*, *V*) to *A*_*I*_. Note that B fails if H_2_List or H_3_List is already defined in the corresponding value, but this only happens with probability smaller than (*q*_2_ + 2*q*_3_ + 2*q*_*sc*_)/2^*k*^.

*O* ^*Designcryption*^ *Queries.* On input (*σ* = (*C*, *U*, *V*), *id*_*S*_, *id*_*R*_), B does the following.

1. If (*id*_*R*_, *PK*_*R*_)≠(*id*_*θ*_, *PK*_*θ*_), B can answer the query according to the specification of the algorithm* Designcrypt* since it knows the receiver *id*_*R*_'s private key and certificate.
2. Otherwise, B searches in H_3_List for all tuples of the form 〈*M*, *U*, *R*_1_, *R*_2_, *id*_*S*_, *PK*_*S*_, *id*_*θ*_, *PK*_*θ*_, *h*_3_, *C*〉. If no such tuple is found, then *σ* is rejected. Otherwise, each one of them is further examined. For a tuple 〈*M*, *U*, *R*_1_, *R*_2_, *id*_*S*_, *PK*_*S*_, *id*_*θ*_, *PK*_*θ*_, *h*_3_, *C*〉, B first checks whether *e*(*H*_1_(*id*_*S*_, *PK*_*S*_)*P*+*P*_pub_, *V*) · (*PK*_*S*_)^−*h*_3_^ = *R*_1_. If the tuple passes the verification, then B returns *M* in this tuple to *A*_*I*_. If no such tuple is found, *σ* is rejected. Note that a valid ciphertext is rejected with probability smaller than *q*_*dsc*_/2^*k*^ across the whole game.

In the challenge phase, *A*_*I*_ outputs (*M*_0_, *M*_1_, *id*_S_*, *id*_R_*), on which it wants to be challenged. If (*id*_R_*, *PK*_R_*)≠(*id*_*θ*_, *PK*_*θ*_), then B aborts. Otherwise, B randomly chooses C* ∈ {0, 1}^*n*^, r* ∈ *Z*_p_*, and V* ∈ *G*, computes U* = r*P, and returns σ* = (C*, U*, V*) to *A*_*I*_ as the challenge ciphertext. Observe that the decryption of C* is C* ⊕ *H*_2_(*e*(U*, *Cert*_*θ*_), *e*(U*,*Cert*_*θ*_)^*SK*_*θ*_^).

In the guess phase, *A*_*I*_ outputs a bit which is ignored by B. Note that *A*_*I*_ cannot recognize that σ* is not a valid ciphertext unless it queries *H*_2_ on (*e*(U*, *Cert*_*θ*_), *e*(U*,*Cert*_*θ*_)^*SK*_*θ*_^) or *H*_3_ on (*M*_*b*_, U*, (*e*(U*, *Cert*_*θ*_), *e*(U*,*Cert*_*θ*_)^*SK*_*θ*_^), *id*_S_*, *PK*_S_*, *id*_*θ*_, *PK*_*θ*_), where *b* ∈ {0,1}. Standard arguments can show that a successful *A*_*I*_ is very likely to query *H*_2_ on (*e*(U*, *Cert*_*θ*_), *e*(U*,*Cert*_*θ*_)^*SK*_*θ*_^) or *H*_3_ on (*M*_*b*_, U*, (*e*(U*, *Cert*_*θ*_), *e*(U*,*Cert*_*θ*_)^*SK*_*θ*_^), *id*_S_*, *PK*_S_*, *id*_*θ*_, *PK*_*θ*_) if the simulation is indistinguishable from a real attack environment. To produce a result, B picks a random tuple 〈*R*_1_, *R*_2_, *h*_2_〉 or 〈*M*, *U*, *R*_1_, *R*_2_, *id*_*S*_, *PK*_*S*_, *id*_*R*_, *PK*_*R*_, *h*_3_, *C*〉 from H_2_List or H_3_List. With probability 1/(*q*_2_ + 2*q*_3_ + 2*q*_*sc*_) (as H_2_List, H_3_List contain at most *q*_2_ + *q*_3_ + *q*_*sc*_, *q*_3_ + *q*_*sc*_ tuples, resp.), the chosen tuple will contain the value *R*_1_ = *e*(U*, *Cert*_*θ*_). Because *e*(U*, *Cert*_*θ*_) = *e*(r*P, *t*(ω* + *α*)^−1^ *P*) = *e*(*P*, *P*)^tr*(ω* + *α*)^−1^^, B returns *T* = *R*_1_^(tr*)^−1^^as the solution to the given *q*-mBDHI problem.

We now derive B's advantage in solving the *q*-mBDHI problem. From the above construction, the simulation fails if any of the following events occurs: (1) *E*_1_: in the challenge phase, B aborts because (*id*_R_*, *PK*_R_*)≠(*id*_*θ*_, *PK*_*θ*_); (2)  *E*_2_: *A*_*I*_ makes an *O* ^*GenerateCertificate*^ query on (*id*_*θ*_, *PK*_*θ*_); (3)  *E*_3_: B aborts in answer one of *A*_*I*_'s *O* ^*Signcryption*^ queries because of a collision on *H*_2_ or *H*_3_; (4)  *E*_4_: B rejects a valid ciphertext at some point of the game.

We clearly have that Pr[¬*E*_1_] = 1/*q*_1_ and ¬*E*_1_ implies ¬*E*_2_. We also already observed that Pr[*E*_3_]≤(*q*_2_ + 2*q*_3_ + 2*q*_*sc*_)/2^*k*^ and Pr[*E*_4_] ≤ *q*_*dsc*_/2^*k*^. Thus, we have that

(4) Pr[¬E1∧¬E2∧¬E3∧¬E4]≥1q1(1−qscq2+2q3+2qsc2k)×(1−qdsc2k).

Since B selects the correct tuple from H_2_List or H_3_List with probability 1/(*q*_2_ + 2*q*_3_ + 2*q*_*sc*_), we obtain the announced bound on B's advantage in solving the *q*-mBDHI problem.

**Lemma 12**

If a Type II adversary *A*_*II*_ has advantage *ε* against our CBSC scheme when asking at most *q*_*cu*_ queries to *O* ^CreateUser^, *q*_*sc*_ queries to *O* ^Signcryption^, *q*_*dsc*_ queries to *O* ^*Designcryption*^, and *q*_*i*_ queries to random oracles *H*_1_ ~ *H*_3_, then there exists an algorithm *B* to solve the BDH problem with advantage

(5) ε′≥εqcu(q2+2q3+2qsc)(1−qscq2+2q3+2qsc2k)×(1−qdsc2k).

**Proof**

Assume that B is given a BDH instance (*P*, *aP*, *bP*, *cP*), where *a*, *b*, *c* are three random elements from *Z*_p_*. B interacts with *A*_*II*_ as follows.

In the setup phase, B randomly chooses *α* ∈ *Z*_p_*, sets *Q* = *aP*, and computes *P*_pub_ = *αP* and *g* = *e*(*P*, *Q*). Furthermore, it randomly chooses an index *θ* with 1 ≤ *θ* ≤ *q*_*cu*_. Then, B starts* IND-CBSC-CCA2 Game-II* by supplying *A*_*II*_ with *msk* = *α* and *params* = {*p*, *G*, *G*_*T*_, *e*, *n*, *P*, *Q*, *P*_pub_, *g*, *H*_1_, *H*_2_, *H*_3_}, where *H*_1_ ~ *H*_3_ are random oracles controlled by B. *A*_*II*_ can make queries on these random oracles at any time during the game.

Now, B starts to respond various queries as follows.

*H*_1_ *Queries*. B maintains a list H_1_List of tuples 〈*id*_*i*_, *PK*_*i*_, *h*_1,*i*_〉. On input (*id*_*i*_, *PK*_*i*_), B does the following: if (*id*_*i*_, *PK*_*i*_) already appears on H_1_List in a tuple 〈*id*_*i*_, *PK*_*i*_, *h*_1,*i*_〉, then B returns *h*_1,*i*_ to *A*_*II*_; otherwise, it returns a random *h*_1,*i*_ ∈ *Z*_p_* and inserts 〈*id*_*i*_, *PK*_*i*_, *h*_1,*i*_〉 into H_1_List.

*H*_2_ *Queries*. B responds as in the proof of Lemma 11.

*H*_3_ *Queries*. B responds as in the proof of Lemma 11.

*O* ^*CreateUser*^ *Queries*. B maintains a list ` of tuples 〈*id*_*i*_, *PK*_*i*_, *SK*_*i*_〉. On input (*id*_*i*_), B does the following: (1) if *id*_*i*_ already appears on KeyList in a tuple 〈*id*_*i*_, *PK*_*i*_, *SK*_*i*_〉, B returns *PK*_*i*_ to *A*_*II*_. (2) Else if *id*_*i*_ = *id*_*θ*_, B returns *PK*_*θ*_ = *e*(*bP*, *Q*) = *e*(*bP*, *aP*) to *A*_*II*_ and inserts 〈*id*_*θ*_, *PK*_*θ*_, ⊥〉 into KeyList. Note that the private key for the identity *id*_*θ*_ is *b* which is unknown to B. (3) Else B randomly chooses *x*_*i*_ ∈ *Z*_p_* as the private key *SK*_*i*_ for the identity *id*_*i*_ and computes the corresponding public key as *PK*_*i*_ = *g* ^*x*_*i*_^. It then inserts 〈*id*_*i*_, *PK*_*i*_, *SK*_*i*_〉 into KeyList and returns *PK*_*i*_ to *A*_*II*_.

*O* ^*ExtractPrivateKey*^ *Queries*. On receiving such a query on *id*_*i*_, B does the following: if *id*_*i*_ = *id*_*θ*_, then B aborts; otherwise, B searches *id*_*i*_ in KeyList to find the tuple 〈*id*_*i*_, *PK*_*i*_, *SK*_*i*_〉 and returns *SK*_*i*_ to *A*_*II*_.

*O* ^*Signcryption*^ *Queries*. On input (*M*, *id*_*S*_, *id*_*R*_), B does the following: if *id*_*S*_ ≠ *id*_*θ*_, B can answer the query according to the specification of the Signcrypt algorithm since it knows the sender *id*_*S*_'s private key and certificate. Otherwise, B randomly chooses *r*, *h*_3_ ∈ *Z*_p_*, *h*_2_ ∈ {0,1}^*n*^ and computes *U* = *r*(*H*_1_(*id*_*θ*_, *PK*_*θ*_)*P* + *P*_pub_)−*h*_3_(*H*_1_(*id*_*R*_, *PK*_*R*_)*bP* + *α* *bP*), *V* = *r* *Cert*_*R*_, *C* = *M* ⊕ *h*_2_, *R*_1_ = *e*(*U*, *Cert*_*R*_), and *R*_2_ = *e*(*U*,*Cert*_*R*_)^*SK*_*R*_^, respectively. It is easy to verify that *e*(*H*_1_(*id*_*θ*_, *PK*_*θ*_)*P* + *P*_pub_, *V*) · (*PK*_*θ*_)^−*h*_3_^ = *e*(*U*, *Cert*_*R*_). It then inserts 〈*R*_1_, *R*_2_, *h*_2_〉 and 〈*M*, *U*, *R*_1_, *R*_2_, *id*_*θ*_, *PK*_*θ*_, *id*_*R*_, *PK*_*R*_, *h*_3_, *C*〉 into H_2_List and H_3_List, respectively, and returns the ciphertext *σ* = (*C*, *U*, *V*) to *A*_*II*_. Note that B fails if H_2_List or H_3_List is already defined in the corresponding value, but this only happens with probability smaller than (*q*_2_ + 2*q*_3_ + 2*q*_*sc*_)/2^*k*^.

*O* ^*Designcryption*^ *Queries*. B responds as in the proof of Lemma 11.

In the challenge phase, *A*_*II*_ outputs (*M*_0_, *M*_1_, *id*_S_*, *id*_R_*), on which it wants to be challenged. If *id*_R_* ≠ *id*_*θ*_, then B aborts. Otherwise, B randomly chooses C* ∈ {0,1}^*n*^, V* ∈ *G*, computes U* = (*H*_1_(*id*_*θ*_, *PK*_*θ*_) + *α*)*cP*, and returns σ* = (C*, U*, V*) to *A*_*II*_ as the challenge ciphertext. Observe that the decryption of C* is C* ⊕ *H*_2_(*e*(U*, *Cert*_*θ*_), *e*(U*,*Cert*_*θ*_)^*SK*_*θ*_^).

In the guess phase, *A*_*II*_ outputs a bit, which is ignored by B. Note that *A*_*II*_ cannot recognize that σ* is not a valid ciphertext unless it queries *H*_2_ on (*e*(U*, *Cert*_*θ*_), *e*(U*,*Cert*_*θ*_)^*SK*_*θ*_^) or *H*_3_ on (*M*_*β*_, U*, *e*(U*, *Cert*_*θ*_), *e*(U*,*Cert*_*θ*_)^*SK*_*θ*_^, *id*_S_*, *PK*_S_*, *id*_*θ*_, *PK*_*θ*_), where *β* ∈ {0,1}. Standard arguments can show that a successful *A*_*II*_ is very likely to query *H*_2_ on (*e*(U*, *Cert*_*θ*_), *e*(U*,*Cert*_*θ*_)^*SK*_*θ*_^) or *H*_3_ on (*M*_*β*_, U*, *e*(U*, *Cert*_*θ*_), *e*(U*,*Cert*_*θ*_)^*SK*_*θ*_^, *id*_S_*, *PK*_S_*, *id*_*θ*_, *PK*_*θ*_) if the simulation is indistinguishable from a real attack environment. To produce a result, B picks a random tuple 〈*R*_1_, *R*_2_, *h*_2_〉 or 〈*M*, *U*, *R*_1_, *R*_2_, *id*_*S*_, *PK*_*S*_, *id*_*R*_, *PK*_*R*_, *h*_3_, *C*〉 from H_2_List or H_3_List. With probability 1/(*q*_2_ + 2*q*_3_ + 2*q*_*sc*_) (as H_2_List, H_3_List contain at most *q*_2_ + *q*_3_ + *q*_*sc*_, *q*_3_ + *q*_*sc*_ tuples, resp.), the chosen tuple will contain the right element *R*_2_ = *e*(U*,*Cert*_*θ*_)^*SK*_*θ*_^ = *e*(*P*, *P*)^*abc*^. B then returns *R*_2_ as the solution to the given BDH problem.

We now derive B's advantage in solving the BDH problem. From the above construction, the simulation fails if any of the following events occurs: (1)  *E*_1_: in the challenge phase, B aborts because *id*_R_* ≠ *id*_*θ*_; (2)  *E*_2_: *A*_*II*_ makes an *O* ^*ExtractPrivateKey*^ query on *id*_*θ*_; (3)  *E*_3_: B aborts in answer *A*_*II*_'s *O* ^*Signcryption*^ query because of a collision on *H*_2_ or *H*_3_; (4)  *E*_4_: B rejects a valid ciphertext at some point of the game.

We clearly have that Pr[¬*E*_1_] = 1/*q*_*cu*_ and ¬*E*_1_ implies ¬*E*_2_. We also already observed that Pr[*E*_3_]≤(*q*_2_ + 2*q*_3_ + 2*q*_*sc*_)/2^*k*^ and Pr[*E*_4_] ≤ *q*_*dsc*_/2^*k*^. Thus, we have that

(6) Pr[¬E1∧¬E2∧¬E3∧¬E4] ≥1qcu(1−qscq2+2q3+2qsc2k)(1−qdsc2k).

Since B selects the correct tuple from H_2_List or H_3_List with probability 1/(*q*_2_ + 2*q*_3_ + 2*q*_*sc*_), we obtain the announced bound on B's advantage in solving the BDH problem.

**Theorem 13**

The CBSC scheme above is EUF-CBSC-CMA secure under the hardness of the q-CAA and CDH problems in the random oracle model.

This theorem can be proved by combining the following two lemmas.

**Lemma 14**

If a Type I adversary *A*_*I*_ asks at most *q*_*cu*_ queries to *O* ^CreateUser^, *q*_*sc*_ queries to *O* ^Signcryption^, *q*_*dsc*_ queries to *O* ^Designcryption^, and *q*_*i*_ queries to random oracles *H*_1_ ~ *H*_3_ and produces a valid forgery with probability *ε* ≥ 10(*q*_*sc*_ + 1)(*q*_*sc*_ + *q*_3_)/2^*k*^, then there exists an algorithm *B* to solve the (*q*_1_ − 1)-CAA problem with advantage *ε*′ ≥ 1/(9*q*_1_).

**Proof**

Assume that B is given a *q*-CAA instance (*P*, *αP*, (*ω*_1_+*α*)^−1^ *P*,…, (*ω*_*q*_+*α*)^−1^ *P*, *ω*_1_,…, *ω*_*q*_), where *q* = *q*_1_ − 1. B interacts with *A*_*I*_ as follows.

In the setup phase, B randomly chooses *t* ∈ *Z*_p_*, sets *P*_pub_ = *αP*, and computes *Q* = *tP* and *g* = *e*(*P*, *Q*). Furthermore, it randomly chooses a value ω* ∈ *Z*_p_* such that ω* ∉ {*ω*_1_,…, *ω*_*q*_} and an index *θ* ∈ [1, *q*_1_]. Then, B starts* EUF-CBSC-CMA Game-I* by supplying *A*_*I*_ with *params* = {*p*, *G*, *G*_*T*_, *e*, *n*, *P*, *Q*, *P*_pub_, *g*, *H*_1_, *H*_2_, *H*_3_}, where *H*_1_ ~ *H*_3_ are random oracles controlled by B. Note that the corresponding master key is *msk* = *α* which is unknown to B.

In the query phase, B responds to various oracle queries as in the proof of Lemma 11.

Finally, in the forge phase *A*_*I*_ outputs a valid forgery (σ* = (C*, U*, V*), *id*_S_*, *id*_R_*) with probability *ε* ≥ 10(*q*_*sc*_ + 1)(*q*_*sc*_ + *q*_3_)/2^*k*^ [29]. If (*id*_S_*, *PK*_S_*)≠(*id*_*θ*_, *PK*_*θ*_), B aborts. Otherwise, having the knowledge of *SK*_R_* and *Cert*_R_*, B runs the algorithm *Designcrypt*(*params*, σ*, *id*_R_*, *PK*_R_*, *SK*_R_*, *Cert*_R_*, *id*_*θ*_, *PK*_*θ*_) to obtain the message M* and then simulates the random oracle *H*_3_ on its own to obtain *h*_3_* = *H*_3_(M*, U*, *e*(U*, *Cert*_R_*), *e*(U*,*Cert*_R_*)^*SK*_R_*^, *id*_*θ*_, *PK*_*θ*_, *id*_R_*, *PK*_R_*). Using the oracle replay technique [29], B replays *A*_*I*_ with the same random tape but with the different hash value *h*_3_^∗′^(≠*h*_3_*) to generate one more valid ciphertext *σ* ^∗′^ = (C*, U*, *V* ^∗′^) such that *V* ^∗′^ ≠ V*. Since σ* = (C*, U*, V*) and *σ* ^∗′^ = (C*, U*, *V* ^∗′^) are both valid ciphertexts for the same message M* and the randomness r*, we obtain the following relations:

(7) V∗−V∗′=(h3∗SKθ+r∗)Certθ−(h3∗′SKθ+r∗)Certθ=(h3∗−h3∗′)SKθCertθ.

Because *Cert*_*θ*_ = *t*(ω* + *α*)^−1^ *P*, B can compute (ω* + *α*)^−1^ *P* = [*t*(*h*_3_* − *h*_3_^∗′^)*SK*_*θ*_]^−1^(V* − *V* ^∗′^) as the solution to the given *q*-CAA problem.

We now derive B's advantage in solving the *q*-CAA problem. From the above construction, the simulation fails after *A*_*I*_ outputs a valid forgery if any of the following events occurs: (1)  *E*_1_: in the forge phase, B aborts because (*id*_S_*, *PK*_S_*)≠(*id*_*θ*_, *PK*_*θ*_); (2)  *E*_2_: B fails in using the oracle replay technique to generate one more valid ciphertext.

Clearly, Pr[¬*E*_1_] = 1/*q*_1_. Moreover, from the forking lemma [29], we know that Pr[¬*E*_2_] ≥ 1/9. Thus, we have that if *A*_*I*_ produces a forgery, then B will succeed in solving the *q*-CAA problem with probability *ε*′ = Pr[¬*E*_1_∧¬*E*_2_] ≥ 1/(9*q*_1_).

**Lemma 15**

If a Type II adversary *A*_*II*_ asks at most *q*_*cu*_ queries to *O* ^*CreateUser*^, *q*_*sc*_ queries to *O* ^*Signcryption*^, *q*_*dsc*_ queries to  *O* ^*Designcryption*^, and *q*_*i*_ queries to random oracles *H*_1_ ~ *H*_3_ and produces a valid forgery with probability *ε* ≥ 10(*q*_*sc*_ + 1)(*q*_*sc*_ + *q*_3_)/2^*k*^, then there exists an algorithm *B* to solve the CDH problem with advantage *ε*′ ≥ 1/(9*q*_*cu*_).

**Proof**

Assume that B is given a random CDH instance (*P*, *aP*, *bP*) where *a*, *b* are two random elements from *Z*_p_*. B interacts with *A*_*II*_ as follows.

In the setup phase, B randomly chooses *α* ∈ *Z*_p_*, sets *Q* = *aP*, and computes *P*_pub_ = *αP* and *g* = *e*(*P*, *Q*). Furthermore, it randomly chooses an index *θ* with 1 ≤ *θ* ≤ *q*_*cu*_. Then, B starts* EUF-CBSC-CMA Game-II* by supplying *A*_*II*_ with *msk* = *α* and *params* = {*p*, *G*, *G*_*T*_, *e*, *n*, *P*, *Q*, *P*_pub_, *g*, *H*_1_, *H*_2_, *H*_3_}, where *H*_1_ ~ *H*_3_ are random oracles controlled by B.

In the query phase, B responds to various oracle queries as in the proof of Lemma 12.

Finally, in the forge phase *A*_*I*_ outputs a valid forgery (σ* = (C*, U*, V*), *id*_S_*, *id*_R_*) with probability *ε* ≥ 10(*q*_*sc*_ + 1)(*q*_*sc*_ + *q*_3_)/2^*k*^ [29]. If *id*_S_* ≠ *id*_*θ*_, then B aborts. Otherwise, having the knowledge of *SK*_R_* and *Cert*_R_*, B runs the algorithm *Designcrypt*(*params*, σ*, *id*_R_*, *PK*_R_*, *SK*_R_*, *Cert*_R_*, *id*_*θ*_, *PK*_*θ*_) to obtain the message M* and then simulates the random oracle *H*_3_ on its own to obtain *h*_3_* = *H*_3_(M*, U*, *e*(U*, *Cert*_R_*), *e*(U*,*Cert*_R_*)^*SK*_R_*^, *id*_*θ*_, *PK*_*θ*_, *id*_R_*, *PK*_R_*). Using the oracle replay technique [29], B replays *A*_*II*_ with the same random tape but with the different hash value *h*_3_^∗′^(≠*h*_3_*) to generate one more valid ciphertext *σ* ^∗′^ = (C*, U*, *V* ^∗′^) such that *V* ^∗′^ ≠ V*. Since σ* = (C*, U*, V*) and *σ* ^∗′^ = (C*, U*, *V* ^∗′^) are both valid ciphertexts for the same message M* and randomness r*, we obtain the following relations:

(8) V∗−V∗′=(h3∗SKθ+r∗)Certθ−(h3∗′SKθ+r∗)Certθ=(h3∗−h3∗′)SKθ(H1(idθ,PKθ)+α)−1Q.

Then, we have the following relations:

(9) e(H1(idθ,PKθ)P+αP,V∗−V∗′) =e(P,(h3∗−h3∗′)SKθQ).

Because *Q* = *aP* and *SK*_*θ*_ = *b*, B can compute *a* *bP* = *SK*_*θ*_ *Q* = (*h*_3_* − *h*_3_^∗′^)^−1^(*H*_1_(*id*_*θ*_, *PK*_*θ*_) + *α*)(V* − *V* ^∗′^) as the solution to the given CDH problem.

We now derive B's advantage in solving the CDH problem. From the above construction, the simulation fails if any of the following events occurs: (1)  *E*_1_: in the forge phase, B aborts because *id*_S_* ≠ *id*_*θ*_; (2)  *E*_2_: B fails in using the oracle replay technique to generate one more valid ciphertext. Clearly, Pr[¬*E*_1_] = 1/*q*_*cu*_. From the forking lemma [29], we know that Pr[¬*E*_2_] ≥ 1/9. Thus, we have that if *A*_*II*_ produces a valid forgery, then B will succeed in solving the CDH problem with probability *ε*′ = Pr[¬*E*_1_∧¬*E*_2_] ≥ 1/(9*q*_*cu*_).

### 5.3. Performance

To evaluate the performance of our new CBSC scheme, we compare our scheme with the previous CBSC schemes in terms of the computational cost and the communicational cost.

In the computational cost comparison, we consider four major operations: pairing, exponentiation in *G*_*T*_, scalar multiplication in *G*, and hash. Among these operations, the pairing is considered as the heaviest time-consuming one in spite of the recent advances in the implementation technique. For simplicity, we denote these operations by *p*, *e*, *m*, and *h*, respectively. In the communicational cost comparison, ciphertext overhead represents the difference (in bits) between the ciphertext length and the message length, |*id*| denotes the bit-length of user's identity, and |*G*| and |*Z*_*p*_| denote the bit-length of an element in *G* and *Z*_*p*_, respectively. Without considering precomputation, the performances of the compared CBSC schemes are listed in Table 2.

The efficiency of a pairing-based cryptosystem always depends on the chosen curve. Boyen [30] computes estimated relative timings for all atomic asymmetric operations (exponentiations and pairings) and representation sizes for group elements when instantiated in supersingular curves with 80-bit security (SS/80) and MNT curves with 80-bit security (MNT/80). In Table 3, we recall the data from [30].

To make a much clearer comparison, Table 4 gives the concrete values of the computational cost and the communicational cost for the compared CBE schemes according to the data in Table 3. As the hash operation is much more efficient than the multiplication in the group *G*, the costs of the hash operations are ignored.

In our proposed CBSC scheme, the* Signcrypt* algorithm does not require computing any time-consuming pairings. It only needs to compute two exponentiations in *G*_*T*_, three scalar multiplications in *G* and three hashes in each signcryption operation. The* Designcrypt* algorithm needs to compute two pairings, two exponentiations in *G*_*T*_, one scalar multiplication in *G*, and three hashes to designcrypt a ciphertext. From Tables 2 and 4, we can see that our scheme is more efficient than the previous CBSC scheme, especially in the computational efficiency. Actually, the computational performance of our scheme can be further optimized when *H*_1_(*id*_*U*_, *PK*_*U*_)*P* + *P*_pub_ can be precomputed. Such a precomputation enables us to additionally reduce one scalar multiplication computation in *G* and one hash computation in both the* Signcrypt* algorithm and the* Designcrypt* algorithm. In addition and most importantly, it is believed that our scheme is the first signcryption scheme in the certificate-based cryptographic setting that achieves security against both the public key replacement attacks and the insider attacks.

## 6. Conclusions

In this paper, we have introduced an improved security model of CBSC that captures both public key replacement attack and insider security. Our cryptanalysis has shown that Luo et al.'s CBSC scheme [20] is insecure in our security model. We have proposed a new CBSC scheme that resists both the key replacement attacks and the insider attacks. Compared with the previous CBSC schemes in the literature, the proposed scheme enjoys better performance, especially in the computation efficiency. However, a limitation of our schemes is that its security can only be achieved in the random oracle model [31]. Therefore, it would be interesting to construct a secure CBSC scheme without random oracles.

## Figures and Tables

**Table 1:** Properties of the related public key cryptosystems.

	Do not require trusted third party	Implicit certificates	Key escrow free	Key distribution free
Traditional PKC	×	×	*✓*	*✓*
IBC	×	*✓*	×	×
CL-PKC	×	*✓*	*✓*	×
CBC	×	*✓*	*✓*	*✓*

**Table 2:** Performance of the CBSC schemes.

Schemes	Signcryption cost	Designcryption cost	Ciphertext overhead
Ours	2*e* + 3*m* + 3*h*	2*p* + 2*e* + 1*m* + 3*h*	2 | *G* |
[18]	1*p* + 1*e* + 4*m* + 3*h*	3*p* + 1*e* + 1*m* + 3*h*	2 | *G*|
[20]	1*p* + 5*m* + 4*h*	4*p* + 2*m* + 3*h*	3 | *G* | +|id|
[21]	2*p* + 4*e* + 3*m* + 3*h*	3*p* + 4*e* + 3*h*	2 | *G* | +2 | *Z*_*p*_|

**Table 3:** Timings needed to perform atomic operations and representation of group elements in bits.

Curves	Relative timings (1 unit = 1 scalar multiplication in *G*)			Representation sizes (bits)	
	*m *	*e *	*p *	|*G*|	|*G*_*T*_|
MNT/80	1	36	150	171	1026
SS/80	1	4	20	512	1024

**Table 4:** Performance comparison of the CBSC schemes.

Schemes	Signcryption cost	Designcryption cost	Ciphertext overhead
MNT/80			
Ours	75	373	342
[18]	187	487	342
[20]	155	602	513 + |id|
[21]	447	594	2390
SS/80			
Ours	11	49	1024
[18]	28	65	1024
[20]	25	82	1536 + |id|
[21]	59	76	3072
